# Blind testing cross-linking/mass spectrometry under the auspices of the 11 ^th^ critical assessment of methods of protein structure prediction (CASP11)

**DOI:** 10.12688/wellcomeopenres.10046.1

**Published:** 2016-12-09

**Authors:** Adam Belsom, Michael Schneider, Lutz Fischer, Mahmoud Mabrouk, Kolja Stahl, Oliver Brock, Juri Rappsilber

**Affiliations:** 1 Wellcome Trust Centre for Cell Biology, University of Edinburgh, Edinburgh, EH9 3BF, UK; 2 Robotics and Biology Laboratory, Technische Universität Berlin, Berlin, 10587, Germany; 3 Institute of Biotechnology, Technische Universität Berlin, Berlin, 13355, Germany

**Keywords:** High-density cross-linking/mass spectrometry, chemical biology, protein cross-linking, protein structure, CASP, structural biology, computational biology, protein modeling

## Abstract

Determining the structure of a protein by any method requires various contributions from experimental and computational sides. In a recent study, high-density cross-linking/mass spectrometry (HD-CLMS) data in combination with *ab initio* structure prediction determined the structure of human serum albumin (HSA) domains, with an RMSD to X-ray structure of up to 2.5 Å, or 3.4 Å in the context of blood serum. This paper reports the blind test on the readiness of this technology through the help of Critical Assessment of protein Structure Prediction (CASP). We identified between 201-381 unique residue pairs at an estimated 5% FDR (at link level albeit with missing site assignment precision evaluation), for four target proteins. HD-CLMS proved reliable once crystal structures were released. However, improvements in structure prediction using cross-link data were slight. We identified two reasons for this. Spread of cross-links along the protein sequence and the tightness of the spatial constraints must be improved. However, for the selected targets even ideal contact data derived from crystal structures did not allow modellers to arrive at the observed structure. Consequently, the progress of HD-CLMS in conjunction with computational modeling methods as a structure determination method, depends on advances on both arms of this hybrid approach.

## Abbreviations

CLMS - cross-linking/mass spectrometry

HD - high-density

NHS - *N*-hydroxysuccinimide

NMR - nuclear magnetic resonance

sulfo-SDA, sulfo-NHS-diazirine, sulfosuccinimidyl 4,4’-azipentanoate

FDR - false discovery rate

MBS - model-based search

HSA - human serum albumin

RMSD - root-mean-square deviation

CASP - Critical Assessment of protein Structure Prediction

Tris - tris(hydroxymethyl)aminomethane

PES - polyethersulphone

IAA - iodoacetamide

LTQ - linear trap quadrupole

MS2 - tandem MS scan

LC-MS - liquid chromatography mass spectrometry

FM - free modelling

## Introduction

Cross-linking/mass spectrometry (CLMS) is a well-established, low-resolution technique for revealing protein interactions in protein complexes and studying protein conformational changes ^1– 16^. In contrast, the use of CLMS to resolve the detailed tertiary protein structure, pioneered by Young *et al.* ^17^, is less well established. A limiting factor is the sparsity of CLMS data. For example, an earlier study from our laboratory found 0.07 links per residue ^18^. A possible culprit for the low data density is the NHS-ester based cross-linking chemistry used in most studies. These cross-linker reagents predominantly react with lysines, which results in linkage maps that are not sufficiently dense to define the detailed structure of proteins.

 In a previous study, we showed that this limitation can be overcome by high-density cross-linking using photoactivatable cross-linkers ^19^. This approach uses a heterobifunctional cross-linker, sulfo-SDA, which on one side carries an NHS-ester and a photoactivatable diazirine on the other side. The diazirine group forms a reactive carbene species upon UV-light activation that can react with any amino acid. The resulting increased data density opens up the possibility of determining tertiary protein structure, which we demonstrated by recapitulating the domain structures of human serum albumin (HSA) in purified form (RMSD to crystal structure of 2.5 Å) and in its native environment, blood serum (RMSD to crystal structure 3.4 Å) ^19^.

 This proof-of-concept study triggered three questions: Would the high-density CLMS (HD-CLMS) method perform robustly on proteins with unknown structure? Would current structure prediction experts be able to improve their predictions using HD-CLMS data? What are the current technical shortcomings of our approach?

To tackle these questions, we embarked on a blind study to evaluate the current capabilities of HD-CLMS in the context of the Critical Assessment of protein Structure Prediction (CASP) experiment. CASP evaluates the state-of-the art in protein modeling ^20^ by the following experiment: Can modeling groups blindly predict the structure of a protein if the structure is unknown to them? Modeling groups predict the structures of these proteins and then have their predictions assessed by independent evaluating scientists.

In CASP11, the Organizing Committee generously offered to identify suitable protein targets for us, which would be sent to our laboratory and give us the opportunity to blindly test our cross-linking method. After putting the proteins through our CLMS pipeline, the Rappsilber lab then submitted CLMS data in the form of distance restraints and these were then offered to the prediction groups as additional data that they could use in their predictions.

Here, we report the outcome of this experiment. In particular, 1) we analyzed whether the blindly generated cross-links fit the crystal structures; 2) we analyzed if CASP modeling groups were able to utilize the CLMS data in their predictions, which is discussed in detail elsewhere ^21^; and 3) we identified technical shortcomings of high-density cross-linking and the blind study approach taken here. Overcoming the issues of HD-CLMS might pave the way for transforming this hybrid approach into a tertiary structure determination method to complement X-ray crystallography, NMR, and cryo-EM.

## Methods

### Proteins

A total of nine proteins were received from five labs. YaaA (PDB|5CAJ) was received from the lab of Prof. Mark Wilson (Department of Biochemistry/Redox Biology, University of Nebraska), as a frozen solution (25 mM HEPES, pH 8.2, 100 mM KCl, 6.89 mg/mL). Five proteins, 413472 (GS13694A), BACUNI_01052 (PDB|4QE0), RUMGNA_02398 (PDB|4QAN), SAV1486 (PDB|4QPV) and BACCAC_02064 (PDB|4QHW), were received from the lab of Dr. Ashley M. Deacon (Joint Center for Structural Genomics (JCSG), Stanford Synchrotron Radiation Lightsource, Stanford University). All were received as previously frozen and thawed-in-transit solutions, with all comprised of a buffer containing 20 mM tris(hydroxymethyl)aminomethane (Tris), pH 7.9, 150 mM NaCl, 0.5 mM tris(2-carboxethyl)phosphine (TCEP) and at concentrations of 2.3, 4.6, 5.2, 2.5 and 11 mg/mL, respectively. MmR495A (no structure in PDB) was received from the lab of Prof. Gaetano Montelione (Center for Advanced Biotechnology and Medicine, Rutgers University) as both a solid lyophilisate (from 20 mM NH4OAc) that had absorbed water during transit and also as a frozen solution on ice containing 10 mM Tris, pH 7.5, 100 mM NaCl, 10 mM DTT, 0.02% NaN _3_. Af1502 (PDB|5A1Q) was received from the lab of Dr. Jörg Martin (Max-Planck Institute for Developmental Biology, Tübingen) as a frozen solution of 30 mM MOPS, 250 mM NaCl, 10% glycerol, pH 7.2, 16 mg/mL. Laminin (PDB|4YEQ) was received from the lab of Prof. Deborah Fass (Department of Structural Biology, Weizmann Institute of Science) as a frozen solution on ice containing PBS and 10% glycerol, 2.4 mg/mL.

Four of the six designated targets (BACUNI_01052, RUMGNA_02398, SAV1486 and BACCAC_02064) were buffer-exchanged prior to cross-linking to remove Tris from the buffer. Buffer exchange was carried out using polyethersulphone (PES) ultracentrifugation devices for concentration of small-volume protein samples, Vivaspin 500, 5000 MWCO, GE Healthcare. Protein concentration was estimated using a Nanodrop 1000 Spectrophotometer from Thermo Fisher Scientific, measuring at 280 nm.

### Chemical cross-linking

Each target was cross-linked using sulfo-SDA, using four different cross-linker to protein ratios (2:1, 1:1, 0.5:1 and 0.25:1, w/w) and four UV activation times (15, 30, 45 and 60 minutes). Cross-linking was carried out in two-stages: firstly sulfo-SDA, dissolved in cross-linking buffer (20 mM HEPES-OH, 20 mM NaCl, 5 mM MgCl _2_, pH 7.8), was added to target protein (30 µg, 1 µg/µL) and left to react in the dark for 1h at room temperature. This allowed the reaction of lysine side chain amino groups but also hydroxyl groups in serine, threonine and tyrosine side chains, with the sulfo-NHS ester component of the cross-linker. The diazirine group was then photo-activated using UV irradiation, at 365 nm, from a UVP CL-1000 UV Crosslinker (UVP Inc.). Samples were spread onto the inside of Eppendorf tube lids by pipetting (covering the entire surface of the inner lid), placed on ice at a distance of 5 cm from the tubes and irradiated for either 15, 30, 45 or 60 minutes. Following the reaction, half of each reaction condition sample was then pooled as a “mixed” sample (a total of 240 µg). The resulting cross-linked mixtures were then separated by electrophoresis using a NuPAGE 4–12% Bis-Tris gel, ran using MES running buffer and stained using Imperial Protein Stain from Thermo Scientific, a Coomassie blue stain. Protein monomer bands were excised from the gel, cut into pieces and then washed to remove Coomassie staining. Proteins were reduced with 20 mM DTT, alkylated using 55 mM IAA and digested overnight using trypsin following standard protocols ^7^. Trypsin/Glu-C co-digestion (in-gel trypsin digestion, overnight at 37 °C followed by addition of Glu-C for 6 hours at room temperature) was used for mixed samples of Target 1 and Target 2. In addition, in-solution Glu-C digestion was used for mixed samples of Targets 2–4. Digests were desalted using self-made C18 StageTips ^22^ prior to mass spectrometric analysis.

### Mass spectrometry and data analysis

Peptides were loaded directly onto a spray emitter analytical column (75 µm inner diameter, 8 µm opening, 250 mm length; New Objectives) packed with C18 material (ReproSil-Pur C18-AQ 3 µm; Dr Maisch GmbH, Ammerbuch-Entringen, Germany) using an air pressure pump (Proxeon Biosystems) ^23^. Mobile phase A consisted of water and 0.1% formic acid. Mobile phase B consisted of acetonitrile and 0.1% formic acid. Peptides were loaded onto the column with 1% B at 700 nl/min flow rate and eluted at 300 nl/min flow rate with a gradient: 1 minute linear increase from 1% B to 9% B; linear increase to 35% B in 169 minutes; 5 minutes increase to 85% B. Eluted peptides were sprayed directly into a hybrid linear ion trap - Orbitrap mass spectrometer (LTQ-Orbitrap Velos, Thermo Fisher Scientific). Peptides were analyzed using a “high/high” acquisition strategy, detecting peptides at high resolution in the Orbitrap and analyzing, also in the Orbitrap, the products of their CID fragmentation. Survey scan (MS) spectra were recorded in the Orbitrap at 100,000 resolution. The eight most intense signals in the survey scan for each acquisition cycle were isolated with an *m/z* window of 2 Th and fragmented with collision-induced dissociation (CID) in the ion trap. 1+ and 2+ ions were excluded from fragmentation. Fragmentation (MS2) spectra were acquired in the Orbitrap at 7500 resolution. Dynamic exclusion was enabled with 90 seconds exclusion time and repeat count equal to 1.

### Data analysis

Mass spectrometric raw files were processed into peak lists using MaxQuant version 1.3.0.5 ^24^ using default parameters, except the setting for “Top MS/MS peaks per 100 Da” being set to 100. Peak lists were searched against a database, comprising in each case only the sequence of the protein that was being analyzed using the cross-linking software Xi ( https://github.com/Rappsilber-Laboratory/XiSearch) for identification of cross-linked peptides. Sequences were provided by the CASP Organizing Committee and are available in the supplement. Search parameters were MS accuracy, 6 ppm; MS/MS accuracy, 20 ppm; enzyme, trypsin; specificity, fully tryptic; allowed number of missed cleavages, four; cross-linker, SDA; fixed modifications, none; variable modifications, carbamidomethylation on cysteine, oxidation on methionine, SDA-loop (SDA cross-link within a peptide that is also cross-linked to a separate peptide). Other SDA modifications (including those resulting from reaction with water and ammonia) were not included in the database search. In earlier work we identified very few such modifications and including these modifications served to increase search database size and also increase false positive identifications, which we were keen to avoid here. Linkage specificity for sulfo-SDA was assumed to be at lysine, serine, threonine, tyrosine and protein N-termini at one end, with the other end having no specificity, i.e. linking to any amino acid residue. A modified target-decoy search strategy was used to estimate FDR ^7, 25^ (Fischer and Rappsilber, unpublished observations ). In short, unique residue pairs are scored on supporting PSMs by:

$$score_{residuepair}=\sqrt{\sum {score_{PSM}}^{2}}$$

Before scoring residue pairs and applying an FDR at their level, the dataset is pre-filtered by applying an FDR-based score cut-off for PSMs and a subsequent FDR-based score cut-off for unique peptide pairs (scored the same way as residue pairs based on supporting PSMs). This provides a means to do noise filtering and can increase the number of unique residue pairs that pass a given FDR. Optimal score cut-offs are automatically defined using xiFDR: https://github.com/lutzfischer/xiFDR.

The MS data have been deposited to the ProteomeXchange Consortium via the PRIDE partner repository with the dataset identifier PXD003643 ^26^.

## Results and discussion

### Outline of the experiment

We had the following objectives for participating in the CASP experiment:

· Test the robustness of high-density CLMS (HD-CLMS)
· Test HD-CLMS driven hybrid methods on difficult protein modeling targets
· Identify methodological shortcomings

**Figure 1.**
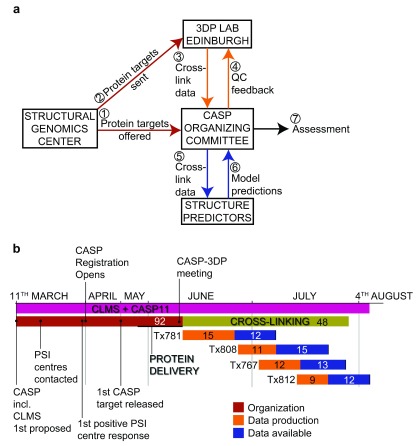
Organization of CASP11 including cross-linking/mass spectrometry. ( **a**) Schematic representation of the interactions between CASP11 participants. ( **b**) CASP11 and CLMS timeline. Total duration of CASP11 and CLMS denoted by the pink bar. Duration of organizational aspects denoted by the red bar. Duration of the cross-linking and mass spectrometry aspect denoted by the green bar. Numbers within the timeline bars denote the number of days in duration for that particular element.

To accomplish these goals, the CASP Organizing Committee identified four CASP target proteins (Tx781, Tx808, Tx767, Tx812, ranging from 204–420 residues in size) whose structures were known to the organizers but neither to our laboratory nor the participating modeling groups. Figure 1 shows the organization and timetable of the experiment.

**Figure 2.**
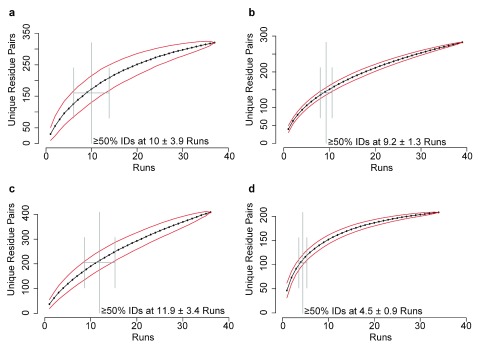
Saturation analysis: Residue pair identifications accumulated over runs. ( **a**) – ( **d**) Total number of unique residue pairs (5% FDR) increases with each successive LC-MS run. The order of LC-MS runs in the series was permutated 100 times and the mean increase per run in all permutations is plotted. ( **a**) Target 1-Tx781. ( **b**) Target 2-Tx808. ( **c**) Target 3-Tx767. ( **d**) Target 4-Tx812.

We cross-linked the CASP11 targets with the photoactivatable cross-linker sulfo-SDA ^19^. We used a panel of different cross-linker to protein ratios and UV activation times to maximize the number of unique cross-links. We digested cross-linked proteins using trypsin and in some cases with trypsin/Glu-C double digestion or Glu-C alone (see Supplemental Text for details). We then subjected the peptide mixture to LC-MS/MS mass spectrometric analysis without an additional enrichment of cross-linked peptides using on average 4.2 days for acquisition. The data were searched against databases derived from the target protein sequences using Xi ^27^. We assess confidence using a target-decoy approach (Fischer and Rappsilber, unpublished observations). We identified cross-links at 5, 10 and 20% FDR and submitted the results to the CASP organizers who made the data available to the modeling groups. At 5% FDR, we identified from 201–381 links for the target proteins. Thus, for the CASP proteins we identified from 0.63–1.2 links per residue, which is comparable to our previous HD-CLMS study on HSA ^19^ ( Table 1). The percentage of links with >11 residues sequence separation, which are most important for protein modeling, were also comparable (59–73% vs 66%, Table 2). Notably, the analysis was not nearing complete detection of linked residue pairs as additional runs kept adding further unique residue pairs, as seen from saturation analysis ( Figure 2). We validated our CLMS data against the crystal structures which became available after the CASP prediction season.

**Table 1.** Acquisition times and cross-link densities at different FDR rate.

Target protein	MS acquisition time	Links per residue at 5% FDR (number of residue pairs)	Links per residue at 10% FDR (number of residue pairs)	Links per residue at 20% FDR (number of residue pairs)
HSA (585 AA)	12 days	0.85 (500)	1.51 (881)	2.56 (1495)
Tx781 (420 AA)	4.7 days	0.73 (305)	1.06 (444)	1.35 (565)
Tx808 (418 AA)	4.4 days	0.63 (265)	0.68 (286)	0.82 (342)
Tx767 (318 AA)	4.0 days	1.20 (381)	n.d.	2.26 (718)
Tx812 (204 AA)	3.8 days	0.99 (201)	1.30 (265)	1.77 (360)

**Table 2.** Number of identified residue pairs >11 residues apart in protein sequence.

Target protein	5% FDR	5–10% FDR	10–20% FDR
HSA (585 AA)	330 (66%) ^1^	292 (77%)	511 (83%)
Tx781 (420 AA)	189 (62%)	100 (72%)	99 (82%)
Tx808 (418 AA)	155 (59%)	15 (71%)	52 (93%)
Tx767 (318 AA)	277 (73%)	n.d.	234 (69%)
Tx812 (204 AA)	146 (73%)	48 (75%)	63 (66%)

^1^Number of links >11 residues apart (percentage of links >11 residues apart of all links)

### Agreement between CLMS data and solved structures

Our first objective is to compare the CLMS data with the crystal structures to test the robustness of high-density cross-linking in a blind experiment.

CLMS experiments do not necessarily reflect the structure of a single conformer but instead reflect the different conformations in the ensemble. Thus, conformational flexibility has to be taken into account when translating the observed cross-links into residue-residue distances. Here we translate observed links into a 25 Å bound on the distance and hence linkable by photo-CLMS using sulfo-SDA, based on a prior analysis of HSA ^19^ (in this study, 25 Å is the distance at which the observed Cα-Cα distance distribution merges with the distribution obtained from decoy matches).

**Figure 3.**
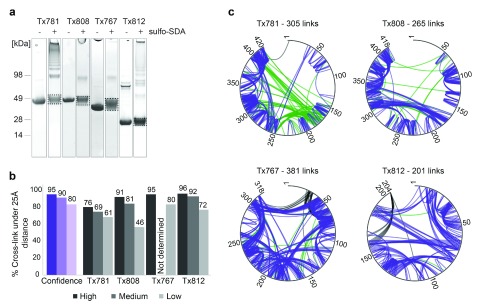
Target cross-linking FDR estimation analysis. ( **a**) CASP11 targets (Tx781, Tx808, Tx767 and Tx812), with (+) and without (-) sulfo-SDA cross-linking. ( **b**) FDR analysis and quality control. FDR estimation on blind data given by the purple columns. Three confidence levels were provided for each target: high (95% true positive hits), medium (90% true positive hits) and low (80% true positive hits). Black and grey columns represent the results of a data QC check by the CASP Organizing Committee, following submission of cross-linking data by 3DP Lab Edinburgh. Numbers on top of black and grey columns represent the percentage of cross-links found in the known crystal structure that had Cα-Cα cross-linking distances of over 25 Å. ( **c**) Cross-link networks for four CASP targets shown for estimated 5% FDR cut-off. Constraints with Cα-Cα cross-linking distances less than 25 Å are shown in purple, constraints with distances 25 Å and over are shown in green and constraints missing from the crystal structure and therefore unverifiable are represented in black.

**Figure 4.**
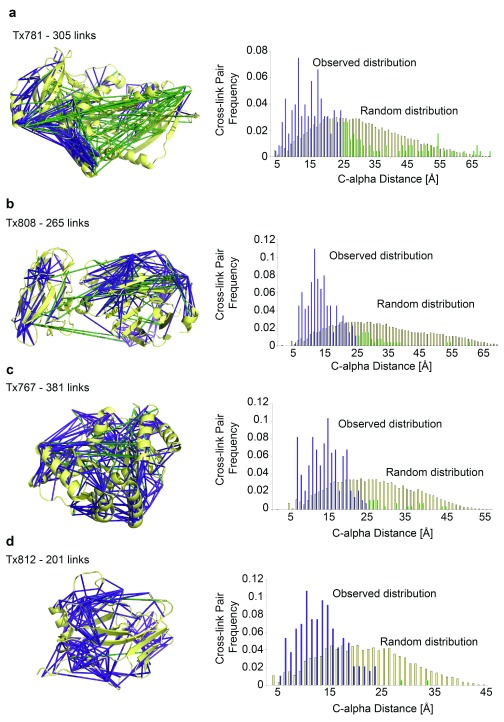
Cross-link distribution within CASP11 Targets. ( **a**) – ( **d**) Left panel shows cross-linked residue pairs at 5% FDR. Right panel shows C-alpha distance distribution of observed constraints at 5% FDR against the random constraint distribution. Constraints with Cα-Cα cross-linking distances less than 25 Å are shown in purple and constraints with distances 25 Å and over are shown in green *.* ( **a**) Cross-linked residue pairs of Tx781 in PDB|4qan, n = 305. ( **b**) Cross-linked residue pairs of Tx808 in PDB|4qhw, n = 265. ( **c**) Cross-linked residue pairs of Tx767 in PDB|4qpv, n = 381. ( **d**) Cross-linked residue pairs of Tx812 in crystal structure (structure not deposited in PDB), n = 201.

With the exception of the first target, Tx781, the 5% FDR lists matched near perfectly to the crystal structures (Tx808 9%, Tx767 5% and Tx812 4% links >25 Å, respectively, averaging to 6% error) ( Figure 3 and Figure 4). This deteriorated slightly when considering 10% FDR data (19%, n.d., 8%; links >25 Å, respectively, averaging to 10.7% error) and further worsened for 20% FDR data (54%, 20%, 28%; links >25 Å, respectively, averaging to 34% error). We attribute the deviation of the computed to the experimentally assessed accuracy to our still small data sets. FDR using the target-decoy approach relies on large data sets. We can see that this condition is not perfectly fulfilled here. For example, in the case of Tx808 data with 20% FDR added only links with very low score to the 10% FDR list, indicating that the data had been exhaustively matched already at 10% FDR. Note that many links are also much shorter than the upper distance bound of 25 Å ( Figure 4).

One explanation for why the crystal structure of Tx781 did not support the CLMS data to the same degree as the other proteins is that the shipping conditions were problematic. The protein arrived defrosted to the laboratory and required buffer exchange from Tris to HEPES. Both might have compromised the integrity of the protein structure. Possibly as a result of this or due to cross-linking, the protein was seen as highly aggregated on SDS-PAGE ( Figure 3a). As a further possibility, the protein may have a different structure in solution from in crystal. Our current data do not allow these possibilities to be distinguished.

A limitation of our current workflow is that we are missing a site assignment scoring and hence do not control for site assignment errors. Importantly, this does not affect the FDR estimation (decoys model well the distribution of long distance and hence likely false links) and we showed in earlier work that we were able to model HSA domains despite site assignment ambiguities ^19^, nevertheless it may falsely elevate the total number of reported links.

In conclusion however, we find good agreement between photo-CLMS and crystallography in this blind study. This suggests that HD-CLMS produces robust residue-residue constraint data.

### Protein structure modeling in CASP11 with CLMS data

Our second objective was to test hybrid modeling methods on our high-density CLMS data, on very difficult modeling targets such as those used in CASP11. The proteins in CASP11 are difficult “free modeling” targets, because even state-of-the-art fold recognition methods are unable to confidently find template structures for template-based modeling. Thus, predictors might use *ab initio* structure prediction, template-based modeling, or a mix of the two approaches.

A full report of this experiment from the protein modeling perspective is published elsewhere ^21^. For the 19 groups that participated in the CLMS-driven and the normal CASP experiment (where no CLMS data is provided), the CLMS data slightly improved the GDT_TS of the predicted models from 36.4 to 38.1 for the first and from 40.9 to 42.0 for the best-of-five submitted models. The GDT_TS is a measure for the match of the prediction to the native structure and ranges from 0 (structures completely dissimilar) to 100 (perfect match). Overall, improvement in modeling accuracy by CLMS-driven predictions is very small and the results suggest no clear improvement.

**Figure 5.**
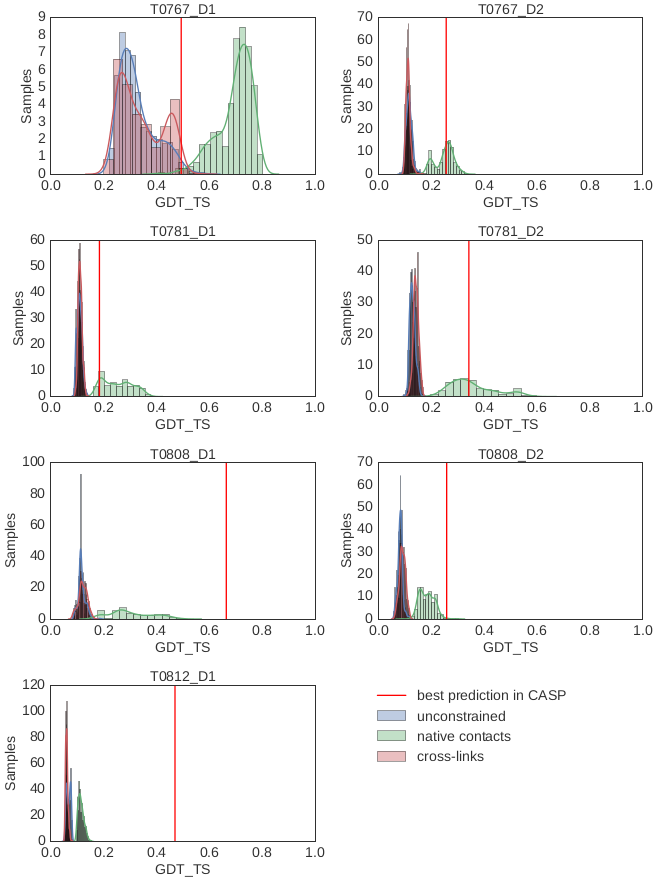
Decoy quality with idealized constraints. Distribution of GDT_TS scores for decoys generated by RBO Aleph without the help of constraints (blue), with cross-links (25Å, red) and native contacts (8Å, green) for the CASP11 cross-linking targets. The GDT_TS of the best model found in CASP11, which also includes template methods is indicated by the red line.

One explanation for this result is that the CASP11 target proteins were too difficult to model, for current state-of-the-art protein structure prediction methods even with accurate residue-residue restraint data. We tested this hypothesis by re-running the modeling experiments with idealized residue-residue constraints taken from the crystal structures ( Figure 5). Note that we evaluated this experiment on the evaluation domains used in CASP11, which are comprised of the partial domains of the full-length protein targets. Even in this idealized scenario, only two out of seven evaluation domains were “foldable” (GDT_TS of the best predicted structural model is 50 or higher). Since the HD-CLMS constraints are inferior to the idealized constraints, both in density and spatial resolution, this data supports our assumption that the CASP11 targets were too difficult to fold for current structure prediction algorithms.

### Identification of methodological shortcomings

Our third objective was to use the CASP experiment to identify any methodological shortcomings of our cross-linking approach. Our analysis of the high-density CLMS data revealed two potential issues with the current method: Uneven sequence coverage caused by uneven distribution of cleavage sites and cross-link bias against β-sheet regions.

**Figure 6.**
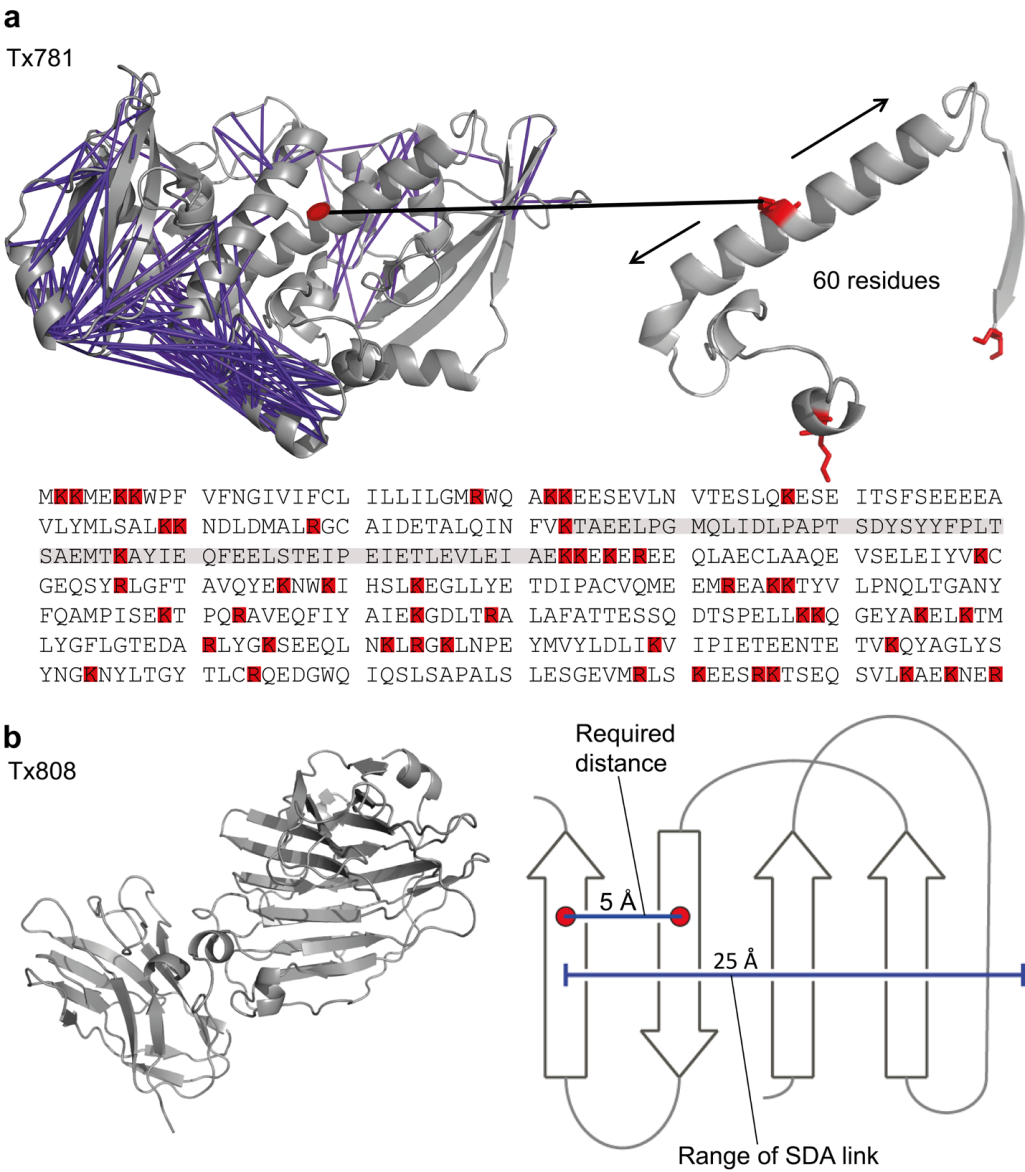
Procedural limitations identified in the study. ( **a**) Top left: Constraints under 25 Å shown in purple, in the crystal structure of Tx781 (PDB|4qan). Top right: Zoom of a 61 amino acid tryptic peptide devoid of observed constraints, containing a single, centrally located lysine residue highlighted in red. Bottom: Amino acid sequence of Tx781. Tryptic (lys and arg) residues highlighted in red. ( **b**) Left: Tx808 crystal structure (PDB|4qhw). Right: Required and actual range of sulfo-SDA to resolve β-sheet topologies.

The issue of uneven sequence coverage was most prevalent for target Tx781 ( Figure 6a). Our analysis of the tryptic digestion sites shows that the absence of observed residue pairs correlates with low frequency of tryptic cleavage sites: up to residue 180 there are 18 tryptic cleavage sites resulting in 0.11 cross-links per residue. For the remaining 224 residues, there were 31 tryptic digestion sites which results in a cross-link density of 0.69 cross-links per residue. The interplay of lysine and arginine residue positions influences whether a cross-linked peptide can be observed for mass spectrometry. For example, the resulting tryptic peptide of cross-linked K126 would be 60 residues long, which is prohibitively long for ordinary mass spectrometry analysis. From the K/R distribution, we estimate a total of 111 residues (62% of the sequence up to residue 180, 26% of the total protein sequence) are thus theoretically inaccessible via trypsin digestion.

We also tested the use of alternative proteases and double-digestion strategies to combat the issue of uneven sequence coverage ( Supplemental Table 1– Supplemental Table 4). Alternative digestion strategies including Glu-C, rather than relying on digestion by trypsin alone increased the number of cross-links ( Table 3). This was particularly striking for the N-terminal domain of Tx781. Glu-C digestion increased the cross-link density from 0.11 to 0.43 links per residue at 5% FDR for the first 180 residues. Thus, our data suggests that alternative or multiple proteases might improve the lack in cross-link density in some regions that is caused by uneven distribution of tryptic digestion sites.

**Table 3.** Number of identified residue pairs added (at 5% FDR) with alternatives to trypsin-only digestion.

Target protein	5% FDR total	Glu-C digestion *^a^*	% Glu-C identified of total
Tx781 (420 AA)	305	119	39%
Of this in N-term. 180 AA	77	57	74%
Tx808 (418 AA)	265	45	17%
Tx767 (318 AA)	381	111	29%
Tx812 (204 AA)	201	36	18%

^*a*^All digestion methods involving use of Glu-C (including trypsin/Glu-C co-digestion (Tx781 and Tx808), and in-solution Glu-C digestion (Tx808, Tx767 and Tx812).

**Figure 7.**
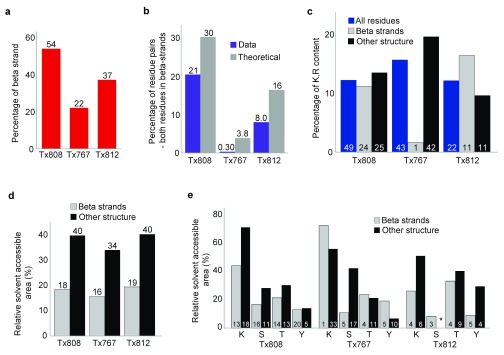
Beta strand analysis for targets Tx808, Tx767, Tx812. ( **a**) Percentage of residues with beta strand structure. ( **b**) Percentage of residue pairs where both residues are beta strand in identified cross-link data compared with the dataset where all possible residue pairs are considered. Residue pairs identified at 5% FDR shown by purple columns. All possible residue pairs with both residues in beta strand shown by grey columns. ( **c**) Percentage of Lys and Arg residues in beta strands versus residues with all other structure types. Percentages of Lys and Arg residues across all residues for each target are shown by blue columns. Percentages of Lys and Arg residues in beta strands for each target are shown by grey columns. Percentage of Lys and Arg residues for all residues, excluding those with beta strand structure are shown by black columns. Numbers within columns refer to the numbers of Lys and Arg found in the crystal structure of each target. ( **d**) Average relative solvent accessible area (RSA) of residues for each target in beta strands compared with average RSA for all other residues, excluding beta strand residues. Average RSAs of beta strand residues are shown by grey columns. Average RSAs of all other residues, excluding beta strand residues, are shown by black columns. ( **e**) Average RSA of Lys, Ser, Thr and Tyr residues for each target, with RSA values of these residues in beta strands compared with values for residues in all other residues, excluding beta strand residues. Beta strand Lys, Ser, Thr and Tyr are shown by grey columns. Lys, Ser, Thr and Tyr from all other residues, excluding beta strand residues, are shown by black columns. Numbers within columns refer to the number of each type of residue found in the crystal structure of each target.

Another issue that we found was an apparent bias against cross-links in β-sheet regions. An extreme example is the target Tx808, where 54% of the residues in the crystal structure have β-sheet structure. The cross-links of this protein are predominantly in the loop region ( Figure 4b). We would expect 80% of the cross-linked residue pairs to have at least one β-strand residue if cross-linking would be unbiased with regard to secondary structure. However, we find only 64% of the cross-linked residue pairs to contain a β-sheet residue (170 of 265; 5% FDR). This β-sheet bias also holds for targets Tx767 and Tx812 ( Figure 7a and b). We exclude Tx781 from analysis because of the issues discussed earlier. We hypothesized that this bias against β-sheet residues might be caused by lack of tryptic cleavage sites, but found that this would only explain the apparent lack in β-sheet cross-links for Tx767 ( Figure 7c). We next considered relative solvent accessibility (RSA) ^28, 29^ and whether this could explain discrepancies in expected and observed cross-link patterns. On average, β-strand residues have much lower relative solvent accessible areas in each of the targets (16–19%) as opposed to other residues (34–40%) ( Figure 7d and e). Consequently, both lack of anchoring residues, cleavage sites and lower RSA may contribute in different ratios and in different proteins to the problem of β-sheet analysis by CLMS. In addition, there is a fundamental issue concerning the use of sulfo-SDA as cross-linker ( Figure 6b). The α-carbon distance between adjacent hydrogen bonded beta strands is in the order of 5 Å ^30^, however the upper limit distance boundary defined by sulfo-SDA is in the range of 20 to 25 Å. This covers as much as five beta strands. Consequently, sulfo-SDA, especially at current data density, is insufficient to reveal the topology of β-strands in a β-sheet.

## Conclusions

Our test of high-density CLMS under the auspices of CASP11 led to three major insights. HD-CLMS can be conducted in under a week and delivers highly reliable structural data on proteins in solution. Nevertheless, while sulfo-SDA proved very useful in solving the structure of the alpha-helical domains of HSA ^19^, HD-CLMS at large may require major developments to achieve a similar success for β-sheet proteins. Sequence coverage and spatial resolution pose a technological challenge and spell out the agenda for future developments. Finally, HD-CLMS is part of a hybrid workflow that relies on structure modeling. Future blind experiments to assess the current value of this hybrid approach need to select protein targets with structures that can be modeled at least when ideal data is available.

## Data availability

The mass spectrometry proteomics data have been deposited in the ProteomeXchange Consortium ( http://proteomecentral.proteomexchange.org) via the PRIDE partner repository with the dataset identifier PXD003643.

## Supplementary material

**Supplemental Tables 1–4.** Zip file consisting of .csv supplemental tables 1 to 4. Tables are made up of lists of LC-MS runs for each of the targets 1 to 4, with the cross-linker:protein ratio and UV activation time used for the sample in each LC-MS run, as well as the protease(s) used for digestion.

**Supplemental Text.** Supplemental Text for "Blind testing cross-linking/mass spectrometry under the auspices of the 11 ^th^ critical assessment of methods of protein structure prediction (CASP11)".

**Protein sequences of Targets 1–4.** Protein sequences of the four target proteins used in the study "Blind testing cross-linking/mass spectrometry under the auspices of the 11 ^th^ critical assessment of methods of protein structure prediction (CASP11)".
