# Supplementary material for: Blind testing cross-linking/mass spectrometry under the auspices of the 11 th critical assessment of methods of protein structure prediction (CASP11)
Source: Wellcome Open Res. 2016 Dec 9;1:24. [Version 1] doi: 10.12688/wellcomeopenres.10046.1 (PMC5354267; doi:10.12688/wellcomeopenres.10046.1)
Supplement: Supplementary file 2 [file wellcomeopenres-1-10823-s0001.tgz › 906ffd64-238d-4c30-a469-aeed99d00a20.docx]

***Detailed description of the experiment***

The pairing of our lab with CASP11 tested the current capabilities of HD-CLMS in the context of a specific set of proteins, selected for being high-level protein structure prediction targets. In addition, CASP participants tested the current value of such experimental data for protein structure prediction. Please note that throughout the entire CASP experiment, the structure of the protein targets was unknown to our group and to the modeling groups.

Structural genomics centers, who are in collaboration with the CASP Organizing Committee, had agreed to withhold the release of their newly resolved structures to the Protein Data Bank (PDB) (Figure 1a). This allowed the blind nature of the experiment to be maintained. The CASP Organizing Committee assessed the suitability of protein structures as potential targets for the CASP11 experiment. Structures were considered for different experiments within CASP11, one of which involved CLMS-assisted structure prediction, known within CASP11 as “contact-guided prediction”. Structures selected for the contact-guided prediction experiment were believed to be particularly challenging for structure predictors. Structural genomics centers providing protein structures, then sent quantities of selected target protein to our lab for cross-linking, data acquisition, data processing and FDR estimation. Cross-link data was then sent to the CASP Organizing Committee which released the data on their website to initiate the second part of the experiment, testing current prediction gains achieved by CLMS data. Both elements of the experiment ended with the release of the respective structures via PDB.

***Detailed experiment time line and target selection***

CASP11 including CLMS was first proposed on March 11^th^ 2014 (Figure 1b). On March 18^th^, the CASP Organizing Committee contacted structure providers to enquire whether proteins could be sent to our lab. The first positive response from a PSI center came 12 days later on March 30^th^. The first proteins arrived to Edinburgh on May 29^th^, with the last proteins arriving on June 9^th^. A meeting was held on June 10^th^ between the CASP Organizing Committee and us to discuss the details concerning the format of the experiment, which of the delivered proteins were to become targets in CASP11 and the order in which data production on the targets was to be done. Cross-linking commenced on June 11^th^. This meant that the organizational phase of the experiment, beginning on March 11^th^, had lasted a total of 92 days. In contrast, protein cross-linking and data processing was carried out for a total of 48 days.

A total of nine proteins (YaaA, 413472 (GS13694A), BACUNI_01052, RUMGNA_02398, SAV1486, BACCAC_02064, laminin, Af1502 and MmR495A) made their way to Edinburgh. One shipment was withheld in customs and arrived defrosted, potentially compromising four targets. Six targets were identified as potential candidates for providing CLMS data. The initial criteria were protein size > 20 kDa, proteins monomeric in solution and having ~1 mg protein sample available (even though we ended up using only 250 µg). We suspected that protein size is an important consideration for CLMS data directed structure modeling, as linked residue pair distance constraints are likely less informative for smaller protein structures, whereas larger proteins are most likely too computationally demanding to model within the time limitations of the experiment. For this reason, Af1502 was discounted as a potential target, having a molecular weight of only 8.3 kDa. The necessity for monomeric proteins was driven by the current requirement that we must avoid cross-linking of homodimeric protein structures, to be certain that observed distance constraints are within a protein structure and not between the monomers of a homodimer. For this reason, MmR495A was discounted as a target. 413472 (GS13694A) was not refined, i.e. no structure was determined and the protein therefore not included in CASP11. A fourth protein to be dismissed, part of the original 6 suitable targets, was YaaA. The protein exhibited total aggregation following cross-linking, according to SDS-PAGE. No attempt was made to optimize the conditions in any way and the target was simply dropped.

Five proteins were taken forward. The expiration dates of these targets were staggered, scheduled for July 1^st^, July 8^th^, July 23^rd^, July 28^th^ and August 4^th^, which dictated the order that the targets were tackled. The first target, Tx781, was due to expire on July 1^st^. Analysis started June 11^th^ and ended June 26^th^ with release of the data. This would have left only 5 days for computation by structure predictors (3 working days), however the PDB agreed to delay the release of this target by a further 7 days, hence the final expiry date became July 8^th^. Protein BACUNI_01052, due to be CASP Target Tx771, was originally scheduled also for release on July 8^th^. After work on Tx781, this left no time to cross-link and acquire data on this target, and for this reason the target was dropped from the experiment. Data was provided for four targets: Target 1, RUMGNA_02398, Tx781; Target 2, BACCAC_02064, Tx808; Target 3, SAV1486, Tx767 and Target 4, laminin, Tx812.

***Detailed Experimental protocol***

As photo-cross-linking is a very recent development as part of CLMS we do not yet possess optimized protocols and a detailed understanding of all factors that govern yield. In a prior analysis of HSA using sulfo-SDA photoactivatable cross-linking (unpublished data), we observed that the number of unique linked residue pairs identified could be increased using different cross-linker to protein ratios and different UV activation times. As CLMS analysis is a stochastic approach, the number of observed residue pairs increases with repeated LC-MS analysis on identical sample. However the maximum number of observable residue pairs in identical sample plateaus after around 4-5 runs. This observation suggests that the limiting factor for identification of unique linked residue pairs is product dependent and the sample has been analyzed exhaustively, within the limits of our chromatography and mass spectrometer. In other words, if we can increase the number of cross-linked peptide products that are in each sample we can increase the unique identifications in each individual run. We therefore mixed individual samples deriving from different cross-link conditions and analyzed them as a “mixed” sample. Furthermore, optimal cross-linking conditions for the CASP targets were not known to us (and most likely would need to be tailored to each target individually) so given the time constraints of the experiment, it made sense for us to pool different conditions. Typically, mixed samples were injected and technically replicated on LC-MS until subsequent injections failed to yield additional unique residue pairs to the data set (typically three injections) (supplemental Tables S1-4).

Bottom-up proteomics approaches rely heavily on the near exclusive use of trypsin, with 96% of deposited data having been generated using trypsin (1). Numerous factors have led to this dominance, but essentially reliance on trypsin means that peptide identifications depend on good sequence coverage of tryptic cleavage residues, Arg and Lys. Cross-linking via amine-reactive NHS-ester based cross-linkers (DSS/BS3/sulfo-SDA) targets Lys residues, which subsequently become non-cleavable by trypsin following cross-linking reaction, the prevalence of Lys and Arg residues becomes even more important (2). It has been shown that cross-linked residue pair identification can be boosted by the use of alternative proteases to trypsin, including proteinase K, Asp-N, Glu-C, Lys-C and Lys-N (2-4). We decided to employ Glu-C to target acidic residues for cleavage for all targets alongside standard trypsin digestion. Trypsin/Glu-C co-digestion was used for Target 1-Tx781 because of the obvious sparseness of tryptic cleavage sites from residues 1-180. The same digestion protocol was used for Target 2-Tx808 but only contributed 15/265 unique residue pairs at 5% FDR (as opposed to 119/305 unique residue pairs at 5% FDR in the case of Target 1-Tx781) so was dropped for remaining targets. For Targets 2-4 (Tx808, Tx767 and Tx812), in-solution Glu-C digestion was used.

The protocol for providing data for CASP11 was identical for each protein target, conforming to a number of steps: (1) buffer-exchange if required; (2) photo-cross-linking, digestion and mass spectrometric analysis; (3) a target-decoy approach FDR analysis and compilation of lists comprising identified residue pairs; (4) submission of our data to CASP11. It should be noted that our data was not posted to the CASP11 participants straight away but subjected to a quality control step by the CASP11 organizers. The CASP11 organizers used their knowledge of the protein structures to verify our confidence intervals (FDR windows) to prevent fundamentally flawed data to be used by the modelers in the second part of the experiment (which was not the case).

We provided the CASP Organizing Committee with linked residue pair lists as .csv files. Three sets of residue pairs were provided in each instance, with 5, 10 and 20% FDR analysis, corresponding to 95%, 90% and 80% residue pair level confidence respectively (Figure 3). We also provided the CASP Organizing Committee with a Perl script (“CASP_Distances.pl”) along with a ReadMe text file “CASP_Distances_ReadMe.txt” in order to check the distances against the crystal structure held by the organizers. Following validation and without any correction, CASP11 organizers released linked residue pair lists to the registered modelling community. Unbeknown to all participants, the organizers only provided those links that were covered by the crystal structure, which fell short of covering the entire protein in all cases. For the four targets for which cross-linking data was actually acquired, there were 12 and 15 calendar days between data being released to the registered modeling community and the expiration of a target (Figure 1b).

***Structure prediction experiments with idealized constraints***

Since using cross-links did not noticeably improve the structure quality, we explored whether the four CLMS cross-linking targets can be folded at all by *ab initio* structure prediction algorithms. To this end, we performed experiments with an ideal constraint set. The constraint set includes all native contacts (residue-residue distances < 8Å in the crystal structure) with a sequence separation of at least 12 amino acids as constraints. This corresponds to the best possible starting point and can be considered as an upper performance bound for constraint-driven structure modeling. The proteins are then folded using these constraints and the RBO Aleph modelling pipeline (5). For comparison, we also folded the proteins without constraints. CASP targets represent very hard modelling cases. If these proteins are at all foldable by current *ab initio* modelling algorithms, the ideal contact set should significantly improve the GDT_TS of the decoys with respect to the native structure. The results are depicted in Figure 5.

**References**

1. Tsiatsiani, L., and Heck, A. J. (2015) Proteomics beyond trypsin. *FEBS J* 282, 2612-2626

2. Petrotchenko, E. V., Serpa, J. J., Hardie, D. B., Berjanskii, M., Suriyamongkol, B. P., Wishart, D. S., and Borchers, C. H. (2012) Use of proteinase K nonspecific digestion for selective and comprehensive identification of interpeptide cross-links: application to prion proteins. *Mol Cell Proteomics* 11, M111.013524

3. Leitner, A., Reischl, R., Walzthoeni, T., Herzog, F., Bohn, S., Förster, F., and Aebersold, R. (2012) Expanding the chemical cross-linking toolbox by the use of multiple proteases and enrichment by size exclusion chromatography. *Mol Cell Proteomics* 11, M111.014126

4. Leitner, A., Joachimiak, L. A., Unverdorben, P., Walzthoeni, T., Frydman, J., Förster, F., and Aebersold, R. (2014) Chemical cross-linking/mass spectrometry targeting acidic residues in proteins and protein complexes. *Proc Natl Acad Sci U S A* 111, 9455-9460

5. Mabrouk, M., Putz, I., Werner, T., Schneider, M., Neeb, M., Bartels, P., and Brock, O. (2015) RBO Aleph: leveraging novel information sources for protein structure prediction. *Nucleic Acids Res* 43, W343-348
