# Supplementary material for: Blind testing cross-linking/mass spectrometry under the auspices of the 11 th critical assessment of methods of protein structure prediction (CASP11)
Source: Wellcome Open Res. 2016 Dec 9;1:24. [Version 1] doi: 10.12688/wellcomeopenres.10046.1 (PMC5354267; doi:10.12688/wellcomeopenres.10046.1)
Supplement: Supplementary file 3 [file wellcomeopenres-1-10823-s0002.tgz › d367d3fd-ff44-4c5f-99ea-56b85b46fb12.docx]

Protein sequences

Target 1 - Tx781, A7B4B4

>tr|A7B4B4|A7B4B4_RUMGN Uncharacterized protein OS=Ruminococcus gnavus ATCC 29149 GN=RUMGNA_02398 PE=1 SV=1

MKKMEKKWPFVFNGIVIFCLILLILGMRWQAKKEESEVLNVTESLQKESEITSFSEEEEAVLYMLSALKKNDLDMALRGCAIDETALQINFVKTAEELPGMQLIDLPAPTSDYSYYFPLTSAEMTKAYIEQFEELSTEIPEIETLEVLEIAEKKEKEREEQLAECLAAQEVSELEIYVKCGEQSYRLGFTAVQYEKNWKIHSLKEGLLYETDIPACVQMEEMREAKKTYVLPNQLTGANYFQAMPISEKTPQRAVEQFIYAIEKGDLTRALAFATTESSQDTSPELLKKQGEYAKELKTMLYGFLGTEDARLYGKSEEQLNKLRGKLNPEYMVYLDLIKVIPIETEENTETVKQYAGLYSYNGKNYLTGYTLCRQEDGWQIQSLSAPALSLESGEVMRLSKEESRKTSEQSVLKAEKNER

Target 2 – Tx808, A5ZGP5

>tr|A5ZGP5|A5ZGP5_9BACE Uncharacterized protein OS=Bacteroides caccae ATCC 43185 GN=BACCAC_02064 PE=1 SV=1

MKNLLCLLALLLCTTVFAQPQQVVVGVSGNGYVTRQQDGARITQRGVTHWTNPKSIVSIYFYLHQPTTADLSLYAKGHSEIKVSYGKKGFKVNLQSNDFTKVPVGSIDIRQAGYVRIDLQGVSKSGEGFGEIKQLIADNVTGKSNYVKDFSDYWGRRGPSVHLGYALPEGDTEWFYNEITVPKEGETMHSYYMAAGFGEGYFGMQYNSPTERRILFSVWSPFDTQNPKEIPDDQKIKLLRQGKDVHIGEFGNEGSGGQSYLKYPWKAGNTYKFLMQIRPDGNGNTTYTAYFYATDEKEWKLIASFLRPKTNTWYKRPHSFLENFSPEQGYLSREVFFGNQWARSKEGKWSRLTDATFTHDATASAQVRLDYQGGNTKDNRFYLKMGGFFNESVPMGTKFYCKPTGKEPEIDWEALKQL

Target 3 – Tx767, A0A0H3JSF1

>tr|A0A0H3JSF1|A0A0H3JSF1_STAAM Uncharacterized protein OS=Staphylococcus aureus (strain Mu50 / ATCC 700699) GN=SAV1486 PE=1 SV=1

MLKKAKLILIAVLVLSGCSTIENESKKDRNTETNTETKSVPEEMEASKYVGQGFQPPAEKDAIEFSKKHKDKIAKRGEQFFMDNFGLKVKATNVVGSGDGVEVFVHCDDHDIVFNASIPFDKSIIESDSSLRSEDKGDDMSTLVGTVLSGFEYRAHKEELDNLTEVLKEYKSKYKYTGYTENAIMKTQNSGFRNEYYYLTAIPYTLDEYKRYFQPLIKEDDKSFRDGMRNSKKQLKDKSRPYVVTTLFSTKDNFTKDNTIDEMIDFSEVLKKKKNIPHDLNVSLQISNKYINTKRPNYSKKEVIEVGVFNHEKANTND

Target 4 – Laminin

>sp|A2B

SEAKGLIRTWVTLKAEQTILPLVDEALQHTTTKGIVFQHPEIVAHMDLMREDLHLEPFYWKLPEQFEGKKLMAYGGKLKYAIYFEAREETGFSTYNPQVIIRGGTPTHARIIVRHMAAPLIGQLTRHEIEMTEKEWKYYGDDPRVHRTVTREDFLDILYDIHYILIKATYGNFMRQSRISEISMEVAEQGRGTTMTPPADLIEK
