# Supplementary material for: Blind testing cross-linking/mass spectrometry under the auspices of the 11 th critical assessment of methods of protein structure prediction (CASP11)
Source: Wellcome Open Res. 2016 Dec 9;1:24. [Version 1] doi: 10.12688/wellcomeopenres.10046.1 (PMC5354267; doi:10.12688/wellcomeopenres.10046.1)
Supplement: Peer review history

# Peer review: Referee response for version 1

**DOI:** 10.21956/wellcomeopenres.10823.r20880

**Published:** 2017-03-13

**Authors:** Mark R. Chance

**Affiliations:** 1 Center for Proteomics and Bioinformatics, Department of Nutrition, School of Medicine, Case Western Reserve University, Cleveland, OH, USA

The manuscript titled “Blind testing cross-linking/mass spectrometry under the auspices of the 11 ^th^ critical assessment of methods of protein structure prediction (CASP11)” by Belsom et al. describes a blind study to test a hybrid approach using cross-linking/mass spectrometry and ab initio modeling (CASP11) in structure prediction. The hybrid approach was proven to be successful on a mostly a-helical protein (HSA) previously published by the group. The new study with 4 proteins from a structural genomics center shows that the CLMS data provide limited or no improvement in modeling accuracy by CASP11. The authors attribute the difference to two major factors. One is the uneven sequence coverage by trypsin (solvable) and the other is the predominant b-sheet structure in the target proteins as opposed to mostly helical structure in HSA. While one of the proteins does have high percentage of the residues in b-sheet (54%), the other two analyzed in the paper have 22 and 37% of the residues in b-strand structure. If the method does not work with these mixed a/b proteins, it will require some major improvement to be effective in structure predication.

 It should be emphasized in fact that the negative results seen here outline the specific milestones that have to be overcome.     

 Other flaws in the manuscript:

1. One protein, TX781, was possibly damaged during shipping, as indicated by the authors. If this is the case, the experiment should be repeated with good protein or the target should be completely excluded from this report.
2. The narrative of the paper is sometimes hard to follow. In the method section, the authors describe in details that a total of nine proteins were received from collaborators, then indicate that 4 out of the 6 designated targets were subjected to buffer exchange to remove tris prior to CLMS. Exactly, how many targets were included in the study?

I have read this submission. I believe that I have an appropriate level of expertise to confirm that it is of an acceptable scientific standard, however I have significant reservations, as outlined above.

# Peer review: Referee response for version 1

**DOI:** 10.21956/wellcomeopenres.10823.r19468

**Published:** 2017-01-18

**Authors:** Jooyoung Lee, Amit Srivastava

**Affiliations:** 1 Center for In Silico Protein Science, School of Computational Sciences, Korea Institute for Advanced Study, Seoul, Korea, South

In their manuscript “Blind testing cross-linking/mass spectrometry under the auspices of the 11 ^th^ critical assessment of methods of protein structure prediction (CASP11)”, submitted for publication in Welcome Open Research, Rappsilber and coworkers have applied the high density cross-linking/mass spectrometry (HD-CLMS) method on four proteins provided by CASP. Using provided cross-linking data, they were able to generate proteins 3D models with slightly improved GDT_TS scores. While the experimental studies appear to be technically solid, authors are asked to answer following comments before indexing of the article.

 Majors: 

1.  Figure 5 shows the distribution of GDT_TS scores for decoys generated by RBO ALEPH without constraints, and with cross links for all four CASP11 cross-linking targets. The GDT_TS of the best model found in CASP11 is also shown in the figure which is relatively quite high for few cases. These results give an impression that the computational method used here is not the best method available because authors claimed that the CLMS data only slightly improved the GDT_TS of the predicted models from 36.4 to 38.1 for the first models and from 40.9 to 42.0 for the best-of-five models.
2. From the viewpoint of generating protein 3D models in the consistent manner with given cross-linking data, authors should perform analysis on all the CASP11 submitted 3D models for 4 Tx targets (7 domains) to observe the correlation between the model’s consistency with cross-linking data and the model’s GDT-TS score. Is the higher GDT-TS score 3D models submitted by other CASP11 groups more consistent with cross-linking data? Or, are there 3D models more consistent with cross-linking data but with lower GDT-TS scores?

Minors:

1. In the data analysis section, what is PSM? How did the author conclude that the residue pair score is equal to square root of PSM score?
2. Both in Table 1 and 2, no data is available for Tx767 for the 10% FDR case. Why?
3. The data set contains only four proteins and the authors did not find good agreement between the photo–CLMS and crystallography data for Tx781. In addition, Tx808 10% FDR data is missing. What is the rationale of the author’s conclusion that “ *we find good agreement between photo- CLMS and crystallography in this blind study”*?
4. What is the reason to choose the upper bound distance of 25 Å? There may exist cross–links with longer than 25 Å and these cross-links data can also improve the results.

We have read this submission. We believe that we have an appropriate level of expertise to confirm that it is of an acceptable scientific standard, however we have significant reservations, as outlined above.

# Peer review: Referee response for version 1

**DOI:** 10.21956/wellcomeopenres.10823.r18753

**Published:** 2016-12-23

**Authors:** Nir Kalisman

**Affiliations:** 1 Department of Biological Chemistry, Hebrew University of Jerusalem (HUJI), Jerusalem, Israel

The manuscript by Belsom *et al.* is an important benchmark in two ways:

1. It generalizes the results of semi-specific cross-linking they obtained last year on a single protein (HSA) to a larger set of proteins. Moreover, these are more challenging proteins with more complex folds. They were also supplied under different conditions from external labs.  The new results clearly show that semi-specific cross-linking is increasing the number of identified cross-links by up to ten folds compared to numbers usually observed by fully specific cross-linking reagents.  This puts HD-CLMS as an important advance for the CLMS methodology.
2. Because the described work was part of the CASP11 prediction experiment, the current effectiveness of the HD-CLMS data for structure prediction is assessed. It is very surprising that the data only improved predictions by very slight amount. In my opinion, the authors have correctly described some of the reasons for this lack of improvement. It highlights the need of the structure prediction community to find better approaches to utilize the data.

 The manuscript is very well written and clear. I have only two minor comments:

 a. In the legend of Fig. 3b – I think the writing should be “cross-linking distances under 25Angs” and not “over 25Angs”.

 b. At the top of page 9 the authors refer to “uncertainty in the site assignment”. I assume it means the uncertainty in the exact residue to which the diazarine is cross-linked to. Perhaps some clarification can be added to the text.

I have read this submission. I believe that I have an appropriate level of expertise to confirm that it is of an acceptable scientific standard.
